# De La Prostitution De La Ville De Paris. M. Duchatelet on the Prostitution of Paris. Second and Concluding Article

**Published:** 1837-01-01

**Authors:** 


### De la Prostitution de la Ville de Paris. By *M. Parent-Duchatelet*, M.D.

[Second Article.]

In our last number we gave a condensed analysis of the first three chapters of the work before us. We find that these volumes, melancholy or even disgusting as they sometimes are, have excited much curiosity among non-professional readers, and we have no doubt that they will do the same within the range of the profession. We shall proceed at once to details.

*Fecundity of Prostitutes.* The state of menstruation varied so much among these females, that nothing very positive could be inferred from investigations the most careful. In respect to fecundity, there is a general impression that prostitutes seldom become pregnant. Inquiries among books and men led to the conclusion that out of a thousand prostitutes scarcely six became annually pregnant, so as to run the full term. They generally have recourse to La Maternité, when about to be confined, and the sage-femme of that establishment, Mad. Legrand, informed our author that from four to six became inmates there every year. They almost all had difficult births, requiring the aid of instruments—and the infants rarely survived. Many of the mothers themselves die in child-bed. Still a more careful and extended enquiry among the medical officers of hospitals, dispensaries, prisons, police bureaus, &c. has convinced our author that pregnancy takes place among prostitutes much more frequently than is imagined. He has reason to believe that, from 30 to 60 are annually delivered in Paris. But abortions during the early months, and even up to seven or eight months of pregnancy, are found to be frequent. Abortions are not seldom procured artificially by these females.

*Syphilis and the Itch.* Of all the diseases to which these poor creatures are exposed, none are more frequent than those which we here mention. But these two are reserved for distinct chapters.

*Menorrhagia—Tumors—Recto-vaginal Fistulæ—Cancer Uteri.* Prostitutes are more subject to suppression of the catamenia than to profuse menstruation. Nevertheless, the latter is not very infrequent. Tumors of the labia pudendi are very common. They are indolent, and chiefly contain a thick albuminous fluid. They grow to an inconvenient size if neglected. When opened they almost invariably discharge a liquid of the most fetid smell. If it touch the hands of the operator, he will not get rid of it for some days. There are about 30 cases annually of recto-vaginal fistulæ, and strange to say, they sometimes heal of their own accord, while these patients are pursuing their ordinary avocations! These fistulæ have generally been found to coexist with phthisis. Cancer of the uterus, though sometimes met with, is very rare among prostitutes.

*Mental Alienation.* Weakness of head—state bordering on insanity—is one of the most frequent complaints that are attributed to the mode of life of the prostitute. Esquirol has received into the wards of the Saltpetrière, an average of 21 insane prostitutes annually—a stupendous proportion! There can be no doubt that this large proportion of mental alienation is owing to the unhappy and unhealthy modes of life pursued by these miserable creatures, and not to mercury, as has been foolishly supposed. Scrofula is exceedingly prevalent among this class also.

### Chap. V.

*Tolerated Brothels.* The police being unable to prevent the existence of such places, tolerates, but does not authorise them. It endeavours to diminish the evils of them. Such haunts have existed in all ages and countries. The Romans designated them *Lupanaria.* In the age of St. Louis (1254) they were christened by the name of Bordeaux in Paris—probably from their being chiefly situated on the borders of the river. They are now called "public houses"—"bad places"—but in the police bureaus—simply "*maisons tolérées*"—tolerated houses. They are under the following regulations. Two of them are not permitted in the same place—that is, with one common entrance, or one common staircase. This wise regulation is grounded on the old maxim—"two of a trade can never agree." Each prostitute is compelled to have a separate chamber to herself. These houses are not permitted to have communication with neighbouring houses—and they must have back or private entrances. Previously to 1811, these places were horrible depôts of filth and sickness. M. Pasquier issued an order to have them all examined, and such as were unwholesome were to be shut up. In fact, this prostitutional reformer made a clean sweep among the brothels, and rendered them comparatively healthy and sweet!

The bagnios are not tolerated in the immediate proximity of churches of any denomination—palaces—great public establishments—the residences of great functionaries—female or male schools—certain furnished lodgings. The distance varies according to circumstances. One hundred paces is the minimum distance of a brothel from a church. Napoleon was very particular on this point—and the Louis, at the restoration, were still more so. Generally speaking, the government sets its face against the establishment of bagnios in narrow and little-frequented streets, where robberies or even murders might be easily committed, in despite of the police.

It is remarkable that rows and "flares-up" are very unusual in the Parisian bagnios. They do take place occasionally, and, as it seems, periodically. They are the result of drunkenness. Saturdays, Sundays, and Mondays, in the low quarters, are the most disorderly.

The keepers of bagnios are allowed to receive *passengers,* as it were—a few beyond the allotted number for which they are tolerated. But there is a number of houses of assignation, which the police has great trouble in watching, and which are exceedingly prejudicial to morals and health. Married women and married men resort to these, and there are hundreds and thousands of spies and emissaries constantly on the look-out for provender for these infernal rookeries! Splendid dinners are given—immense sums gambled away—and the most tip-top prostitutes are there to be found! But we must pass over a number of details which are interesting to the inhabitants of Paris, but not to us.

### Chap. VI.

*Registry or Inscription of Prostitutes.* This was commenced in the French capital in 1765, but the records are either lost or useless. In the storms of the revolution, the prostitutes were left to their own governance, and the most scandalous scenes of debauchery were daily presented to public view. It was not till the year 1796, that some kind of order was re-established—and then the order was little more than disorder. In 1816, the system assumed a considerable degree of perfection—and in 1828, it was brought to its present state of discipline.

INSCRIPTION.

The inscribers are ranged in three classes.

1mo. Those who come to the office, and register their own names.

2ndo. Those who are brought to the office by the keepers of houses.

3tio. Those who are arrested by the inspectors and brought to the office.

In all these cases certain interrogatories are put. The name, age, place of birth, profession, present residence, are all registered. Then she is asked if she be married, widow, or single—if her parents be living, and how employed—why she quitted them, if she have quitted her family—whether or not she has had children—how long in Paris—how long in a state of prostitution, if she have commenced the trade—if she has had, and how often, any venereal complaint—if she has received any education—why she has determined to register?

These and numerous other interrogatories fail not to draw forth a great deal of curious and valuable information. They are visited by the deputed physician, and certified as to soundness or unsoundness. The neophyte then subscribes to certain conditions, thus forming a kind of contract, which legalizes the punishments which are often obliged to be inflicted on them afterwards. Not long ago three elegant young English ladies, of superior education and polished manners, applied at the Bureau to inscribe their names. The officer was convinced that there was some secret kept in the back-ground, delivered them passports, and caused them to quit Paris immediately. Young people coming to register and declaring themselves to be virgins are refused a licence.

A long description is given by our author of the mistresses or keepers of houses of ill-fame, and the qualifications which they must possess, before they are granted a license. They must not be too young—they must prove that they are possessed of some property, &c. but they contrive to elude most of the conditions imposed on them. These dames have no small opinion of their consequence. They do not consider *their* trade as at all illicit or disreputable. They think themselves as much above the prostitutes by whom they live, as the great landowners in this country consider themselves elevated above the boors on their estates. They have a secretary, with a large salary, who manages all their literary concerns, petitions, &c. &c. He has placed over his Bureau—" *this is the grave of all secrets.*" The author has recorded a number of petitions from these dames, and from prostitutes who are candidates for the honourable and lucrative office of brothel-keeper, but these we must pass over. In the recruit of their corps they evince considerable skill, without many scruples. They have agents in the hospitals—and even in the country, to entice fresh hands into their unhallowed vineyard. Our author describes the state of dependence, or rather slavery, in which the mistresses of these houses hold their unfortunate inmates! This, however, we believe, is not peculiar to Paris, but applies to all capitals. One good trait in the character of these dames is, that they never allow their own children to be brought up in their own houses. They generally indeed give them a good education, and have been known to advance large sums to get their children well off in the world. They take the greatest pains to conceal from their offspring the nature of their own avocations.

Clandestine Prostitution is next delineated in its horrible effects on public morals and private health. He considers it infinitely more destructive to both than public prostitution. The most infamous feature in this dark conspiracy against virtue and morality, is the seduction of mere children to be sold at a high price to rich debauchees! The schemes and manœuvres practised for these purposes are too heart-rending for detail, and we therefore pass them over.

The second volume and the 15th chapter commence with a curious subject and a curious question—what becomes of prostitutes when they are no longer able to pursue their triste avocations? This is not easily answered. We have often asked ourselves what becomes of the bodies of all the wild animals that die on this earth? It is somewhat the same with the prostitutes—Nobody knows! It may be remarked, however, that some of them realize a little property, on which they retire after a certain age—some get married—others are received back by their friends—some procure service—many are imprisoned, or get into hospitals or other establishments where they die. A majority, however, of these unfortunates disappear from the stage of existence in a manner totally unknown to the Police!

The 16th Chapter is on the benefit—the necessity of keeping a sanitary surveillance over prostitutes, and of examining them from time to time, in order to check syphilis—a disease worse, in M. P.'s opinion, than the plague and all other contagious maladies. It was in 1747 that the idea of examining prostitutes was first started; but it was not till 1802, that a kind of prostitute medical club was established, under no less authority than M. Leroux, who pocketed six thousand francs annually—for doing nothing. The demerits of this club (supported by a tax on the prostitutes) were at length denounced, and the "Dispensaire de Salubrité" was re-organized and reformed under the auspices of M. Pasquier in 1810. The new Personnel was composed of a first surgeon and a first physician—a sub chief physician, and ditto surgeon, at 3000 francs a-year each, the higher ranks having double that sum—two eleves, one medical, the other surgical, at 1800 francs each—an apothecary and registrar, at 2400 francs—in all 24,000 francs per annum. M. Pasquier ordered a weekly report to be regularly transmitted to him from the Dispensary—and a permanent commission was formed to watch over all its concerns.

Under this new regime, the frightful extent of venereal diseases was first clearly recognized, and great hopes were entertained that a stop would be put to the spread and even existence of the loathsome malady, when the invasion of France by the allies, threw every thing into disorder in 1814, and the same confusion prevailed in 1815, after the battle of Waterloo. The prodigious number of foreign troops, at these epochs, in Paris and its vicinity, augmented the amount of syphilitic affections to an inconceivable extent. M. Anglès came to the Prefecture, and eclipsed even M. Pasquier, in vigilance and correction of the evil. This good state of things did not last long; for we find from 1820 to 1828, every thing went into disorganization. The reason of this is not satisfactorily given.

The sanitary visits of the officers of the Dispensary are made at three different places—viz. at the Dispensary itself—at the houses of the females—and at the Depôt of the Police. At the Dispensary are seen, twice a month, the free girls (filles libres), all those who are registered for the first time—and all those who, wishing to leave Paris, demand a passport for that purpose. The examination is made in a private apartment. In the prisons and hospitals the inspection takes place on a table, similar to that on which operations are performed; but at the Dispensary, this is not done, lest the bonnets of these "elegantes" should be hurt by the recumbent posture. On a moderate calculation each medical officer can examine twenty-five prostitutes per hour, and enter their report in a book.

The domiciliary inspections are made twice a month at the houses of the individuals, and registries kept of all diseases with which the women may be affected. An infected girl is immediately ordered to the Dispensary, where she is again examined, and despatched to the hospital. The number of infected females sent to hospitals monthly, varies from 51 to 110—and the distribution of infection, before these are sent to the place of cure, must be enormous. In times of prosperity and plenty the number of venereal affections always increases, and *vice versa*. When the cholera raged, venereal affections almost disappeared!

There are a certain, or rather uncertain number of prostitutes who are insusceptible of the contagion of syphilis. This is incontestible; but, in this respect, they differ not from men, a certain proportion of whom are also infractory (to use a Gallic expression) to venereal maladies. Some of the physicians attached to the dispensaries believe that one-half of the prostitutes are insusceptible of the venereal disease. Our author thinks that they have over-rated the proportion of insusceptibles, but they are not a great way from the truth. He gives strong reasons for this conclusion, into which we cannot enter. We do not know that an exact calculation would be of any particular use; but the immunity in question may be serviceably applied to the immunities from other infectious diseases—and still more to epidemics.

Notwithstanding the ability acquired by the dispensary physicians of detecting diseases in the prostitutes submitted to their examination yet they are sometimes at a loss, and cannot decide. They then put the suspected person in sequestration, and order the mistress of the brothel to keep her from communication with man till she is again examined, under pain of having the establishment closed for a longer or shorter time.

Has the intensity and frequency of venereal affections abated within the present century? This important question is decided in the affirmative by our author. Unfortunately the statistical tables by which he meant to prove this affirmative, were not constructed at the period of his decease, and we have only his own assertion in proof of the fact. Fortunately for humanity all practical observers concur in the truth of the assumption that venereal diseases are infinitely milder in their nature, as well as less frequent in their occurrence, than in the days of our ancestors. M. P. Duchatelet affirms that there is a pretty regular decennial decrease in the virulence and number of syphilitic maladies.

Our author endeavoured to ascertain whether *age* had any influence in modifying the susceptibility to venereal infection, but he acknowledges that his enquiries have not led to any satisfactory result. The same observation applies to the season of the year. M. Trail, of Nancy, however, asserts that, from thirteen years of observation and registry, chancres and buboes are more frequent in Winter—gonorrhœa in Spring and Summer.

### HOSPITALS FOR VENEREAL DISEASES.

The venereal disease was first recognized in Paris, on the 6th March, 1497, as proved by an "Arrêt" of Parliament, which ordered all infected foreigners to return to their own countries—people of easy circumstances to keep within their own houses—and the poor to repair to a certain establishment allotted for their reception. No means of cure entered into the regulations of this venereal hospital—which, by the way, was only for males, the females being left to their own resources. It was not till the year 1505, that an hospital for the treatment of the disease was *ordered* by Parliament—which order remained a dead letter for 30 years afterwards! In 1535 a building was erected containing two wards—still for males—capable of containing between three and four hundred patients. But various intrigues rendered this establishment completely inefficient, and it was not till the year 1614, that the Hôtel Dieu was compelled to afford succour to venereal patients—and these only the lords of the creation! Not a word was ever heard of any establishment or remedy for females! In 1657, in the reign of Louis the 14th, some indication of affording relief to infected females was apparent—and but apparent! In 1691, venereal females were admitted into the BICETRE, where the state of these wretches baffles all description, as to misery and disease! In 1730, there were collected, in a narrow ill-ventilated, damp, and crumbling corner of the BICETRE, 400 syphilitics. They were, for the most part covered with ulcers, and in the most deplorable state of health. In 1784, M. Breteul, Minister of the Interior, was horrified at visiting this depôt of misery and vice, and projected a syphilitic hospital in the Convent of the "Capucins de St. Jaques," but like many other projects, it fell to the ground. The Constituent Assembly took the affair in hand, and since 1792 the venereal wards of the CAPUCIN have presented a very different aspect.

The venereal hospitals were sadly deranged in 1815, when France was invaded; but since that period, their organization has been more complete than ever. There are now several. In some of them, as the HÔPITAL DU MIDI, the patients have been set to work, in making charpee, &c. &c. which project has completely failed. Religious instruction was tried with much beter success. The prostitutes attended regularly at divine service, and behaved themselves in the most respectful manner.

Several chapters on police regulations and other matters connected with Parisian prostitution must be passed over in silence, as not particularly interesting to the English medical reader. We close the book with considerable regret that the author did not avail himself of the unique opportunities which his situation afforded him, of making statistical reports on the more purely professional part of the subject. The state of medicine and surgery, as far as regards the various forms of venereal affections, is entirely passed over. We can easily believe, however, that, had the author lived, this subject would have been fully treated of. Death cut him short—and the present volumes only were left fit for publication. They will be read very extensively beyond the sphere of the medical profession, and they will remain a lasting memorial of the author's unexampled industry. We agree with a contemporary reviewer, that there is scarcely a sentence in these volumes calculated to offend the most delicate ear, notwithstanding the revolting nature of the subject! We are not sure that a considerable portion of these volumes might not be perused in the *boudoirs* of our ladies, without any serious injury—perhaps with advantage. But, be that as it may, the politician, philosopher, philanthropist, and physician, will read these volumes with no common interest. In the two articles which we have dedicated to their analyses, we have endeavoured to present the chief points of interest to a large class of our readers who cannot be expected to purchase or peruse the original.

